# Neuromuscular Magnetic Field Measurement Based on Superconducting Bio-Sensors

**DOI:** 10.3390/mi14091768

**Published:** 2023-09-15

**Authors:** Zhidan Zhang, Anran He, Zihan Xu, Kun Yang, Xiangyan Kong

**Affiliations:** 1 The Institute for Future Wireless Research (iFWR), Ningbo University, Ningbo 315211, China; 2101100023@nbu.edu.cn (Z.Z.); 2111082279@nbu.edu.cn (A.H.); 2201100027@nbu.edu.cn (K.Y.); 2 The Faculty of Electrical Engineering and Computer Science, Ningbo University, Ningbo 315211, China

**Keywords:** SQUID, neuromuscular magnetic field, noise suppression, environmental compensation, modeling

## Abstract

These years, disease-causing and disabling diseases have caused great concern. Neurological musculoskeletal disorders are diverse and affect people of a wide range of ages. And the lack of comprehensive diagnostic methods places a huge burden on healthcare systems and social economies. In this paper, the current status of clinical research on neuromuscular diseases is introduced, and the advantages of magnetic field measurement compared with clinical diagnostic methods are illustrated. A comprehensive description of the related technology of superconducting quantum interference devices (SQUIDs), magnetic field detection noise suppression scheme, the development trend of the sensor detection system, and the application and model establishment of the neuromuscular magnetic field is also given in this paper. The current research and development trends worldwide are compared simultaneously, and finally the conclusions and outlook are put forward. Based on the description of the existing literature and the ideas of other researchers, the next development trends and my own research ideas are presented in this paper, that is, starting from the establishment of a neuromuscular model, combining medical and industrial work, designing a sensor system that meets clinical needs, and laying the foundation for the clinical application of a bio-magnetic system. This review promotes a combination between medicine and industry, and guides researchers on considering the challenges of sensor development in terms of clinical needs. In addition, in this paper, the development trends are described, including the establishment of the model, the clinical demand for sensors, and the challenges of system development so as to give certain guidance to researchers.

## 1. Introduction

Unlike heart- and brain-related diseases, which carry a higher risk of death, bone-, muscle- and nerve-related diseases have received relatively little attention in the past. However, with the improvement in living standards, disease-causing and disabling diseases have gradually received attention. In addition, diverse musculoskeletal diseases cause a heavy medical burden in every country, and the burden seriously exceeds the service capacity [1,2]. Young children and adolescents are both at risk of musculoskeletal diseases [3]. In China, muscle nerve diseases, such as chronic non-specific low back pain, due to their complexity and the lack of correct diagnostic methods, also bring a huge burden to the medical system [4].

A survey report of 354 diseases in 195 countries, including China from 1990 to 2017, pointed out [5] that skeletal neuromuscular diseases accounted for a relatively large proportion and showed an increasing trend. A summary report on the impact of skeletal muscle disorders in the United States [6] states that skeletal and muscle disorders are systemic and very common, with one in two people being diagnosed with musculoskeletal disorders. In addition, musculoskeletal diseases affect human normal life and economic development [7,8].

When musculoskeletal disorders that can be prevented or improved are not addressed in a timely manner, opportunities to intervene earlier and more effectively in the disease are missed, and the problem is made worse by the lack of methods for effective diagnosis. Therefore, a clinical detection method is urgently needed to improve the accuracy of diagnosis. At present, there are a variety of diagnostic methods in clinical practice, such as electromyography (EMG) [9,10], surface electromyography [11], needle electromyography [12,13], quantitative electromyography [9], ultrasound [14], magnetic resonance imaging (MRI) [15], etc. But they have their own advantages and disadvantages. For EMG, the subcutaneous tissue is similar to low-pass filtering, and the human body conducts electricity, which will affect the transmission of electrical signals and reduce the accuracy of signals [10]. Needle EMG test signals are accurate but invasive, and the location of the needle depends on the doctor’s clinical experience [13]. Therefore, a method is needed to combine with other diagnostic methods to improve the accuracy of clinical diagnosis and solve some problems in the diagnosis of neuromuscular diseases. The electric field and magnetic field are homologous. The muscle action potential is accompanied by electrical activity, and at the same time, it will radiate in space in the form of a weak magnetic field, so disease diagnosis by detecting magnetic field is completely non-invasive.

In 1972, David and Edward used a superconducting quantum interference device (SQUID) to measure the muscle magnetic signal for the first time in a magnetic shielding room and defined it as a magnetomyogram (MMG) [16]. Related instruments for the detection of magnetic signals in the heart and brain have gradually matured and entered the clinic [17,18,19]. However, due to the difficulty of muscle nerve signal detection, the clinical application of MMG for disease diagnosis has been slow to develop. The clinical diagnostic technology and application of MMG in China are still lacking. SQUID has been used to conduct preliminary testing and analysis of muscle signals at the laboratory for the first time in China [20], and more relevant experiments are currently being carried out.

Some international research teams have pointed out that the neuromuscular magnetic field has potential advantages in disease diagnosis, health detection, rehabilitation and robot control [21,22]. Compared with EMG, MMG, with a higher signal-to-noise ratio, non-invasive property, higher signal accuracy, and being insensitive to surrounding muscle tissues can simultaneously measure multiple dimensions for source localization [23]. The magnetic detection of spinal nerves has certain advantages compared with other clinical detection methods [24]. In addition, MMG has potential advantages in providing additional details about the mechanism of skeletal muscle contraction [25]. Therefore, the magnetic detection of nerve and muscle is expected to become a new auxiliary detection technique, which is of great significance in the clinical diagnosis of diseases and the study of the kinematic mechanism. The advantages and disadvantages of EMG, MRI and MMG are shown in Table 1.

## 2. System-Related Technology

**Figure 1:**
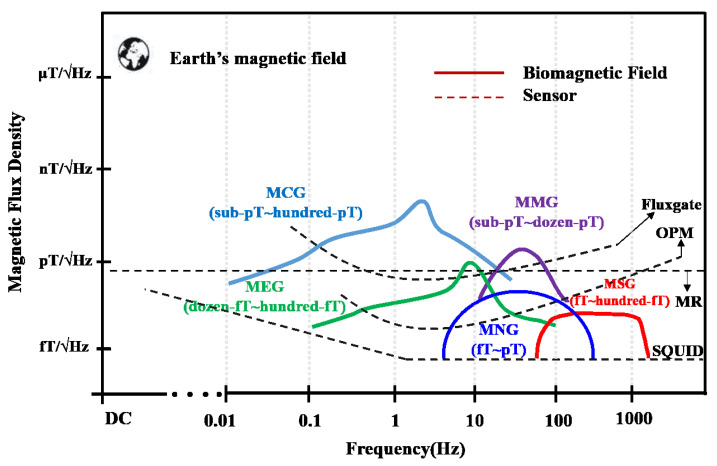
Biomagnetic signals characteristics and sensor sensitivity relationship.

Magnetoneurography (MNG), MMG, magnetocardiogram (MCG) and magnetoencephalogram (MEG), relative to the Earth’s magnetic field, are very weak magnetic field signals. To detect such a weak magnetic signal requires highly sensitive magnetic field sensors. SQUID are sensors that meet the detection requirements in sensitivity, bandwidth and time response. Optical pumped atomic magnetometers (OPMs) are limited by their bandwidth and cannot meet all neuromuscular magnetic field signal tests, but they can detect the magnetic field signals of some muscles or nerves [26,27,28]. Other sensors, such as fluxgate and reluctance sensors, cannot meet the detection requirements at present. The sensors include their detection sensitivity and working bandwidth, and are shown in Figure 1 [29,30]. Comparing the different approaches to detecting the bio-magnetic field, due to the sensitivity and size of OPM, it has been a concern of scientists in recent years. However, as can be seen from Figure 1, SQUID is currently the most suitable sensor for all nerves and muscles detection. It is still the gold standard for biological magnetic field detection, so in the laboratory, SQUID must be used for abundant experiments to establish detection standards of the MMG, MNG, and MSG. But for clinical diagnosis and even daily personal physical examination in the future, reluctance sensors should have the greatest advantages, which do not need high prices and can achieve array miniaturization.

### 2.1. Sensor-Related Technology

SQUID [31] combines the two physical phenomena of magnetic flux ionization and Josephson effect, which is the embodiment of quantum behavior at the macro level, and is also the magnetic field sensor with the highest sensitivity theoretically so far. In order to improve the performance of SQUID, different research teams at home and abroad have focused on the quality of the Josephson junction that makes up SQUID. The critical current density of the Josephson junction is further increased and the junction size is further reduced. Table 2 shows the research status of the Josephson junction of different teams in recent years. By optimizing the performance of a single junction, researchers can use it for many applications, such as NanoSQUID [32], SFQ [33], TES [34], etc. For the actual biomagnetic detection, the magnetometer, gradiometer and current sensors are the core detection elements. Table 3 shows the research status of different SQUID-sensitive elements.

**Figure 2:**
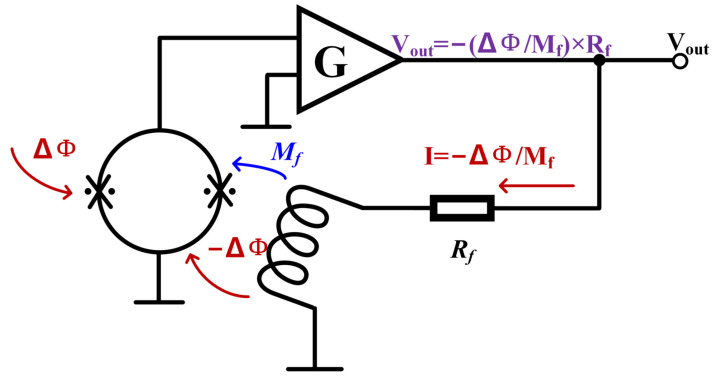
The principle of FLL.

**Figure 3:**
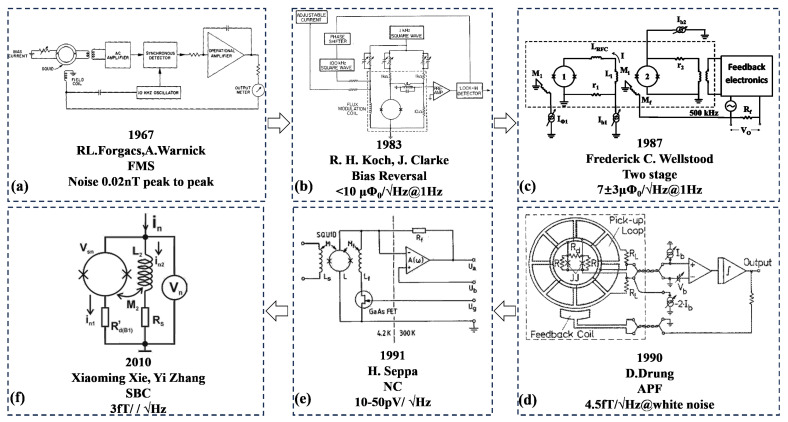
Development of SQUID readout circuit. (**a**) Flux modulation scheme (FMS ) [47]; (**b**) bias reversal circuit [49]; (**c**) two-stage SQUID circuit [50]; (**d**) additional positive feedback (APF) [51]; (**e**) noise cancellation (NC) [52]; (**f**): SQUID bootstrap circuit (SBC) [53].

The output of SQUID is modulated by an external magnetic flux in a periodic way, but the output is not linear to the detected magnetic field and cannot be directly used for magnetic field measurement. A specific readout circuit is needed to improve the dynamic range of the sensor’s magnetic flux so that the output and magnetic flux present a linear relationship. The key structure of this circuit is the flux-locked loop (FLL) [47,48]. The basic principle is shown in Figure 2. It uses a negative feedback circuit to generate a magnetic flux equal to and opposite to the external change on the feedback coil to make the sensor work in a fixed state, called the working point, and then achieves a linear readout. Since the appearance of FLL, researchers have invented a variety of different readout circuits but they are all based on FLL. Figure 3 shows the development history of the SQUID readout, and their starting point is to achieve a higher signal-to-noise ratio and a more stable signal readout. Table 4 shows the performance of the readout circuits used by different research teams, which are used in different applications and, therefore, different parameters are focused on. However, to read out weak signals, it is of importance for the matching between the sensors and its readout circuit, and a better test environment and a stable working platform are also in need so that the advantages of SQUID can be played, and the development of the sensor in the application can be promoted.

### 2.2. Environmental Assessment and Noise Suppression Methods

Compared to the Earth’s magnetic field of 30–50 μT, the intensity of the space magnetic field generated by bioelectrical activities, such as neuromuscles, is extremely small. Therefore, before the actual signal test, it is necessary to evaluate the environment, select different noise suppression means and achieve the suppression of the environmental magnetic field. Among them, sensors with low sensitivity, such as fluxgate [60], can be used to test magnetic field fluctuations in the time domain and gradient field fluctuations, and the frequency domain characteristics of the environmental field can be tested by a SQUID magnetometer with low sensitivity. If the sensor is in a shielded room, it is also necessary to evaluate the shielding effect of the shielded room.

After evaluating the signal, it is necessary to select different noise shielding and signal detection schemes according to the environmental characteristics and the amplitude and frequency domain characteristics of the detected signal. Noise shielding is mainly divided into active shielding and passive shielding [61]. Passive shielding is the use of high-permeability materials, such as permalloy, to build a shielding room, shielding cylinder, etc., which can play a certain shielding role in the environment of remanence and the gradient field. Depending on the number of layers, the shielding effect is also different. Table 5 shows passive shielding schemes developed by different research teams.

Although passive shielding is costly and requires a large space, open magnetic shielding rooms may become a possibility for future development in the medical field. At present, due to the high cost of passive shielding, the development of active shielding has become the mainstream. Active shielding mainly captures the magnetic field signal through the magnetic field sensor, and passes the signal into the signal source so that it generates a certain current and passes into the Helmholtz coil to generate a magnetic field equal to the ambient magnetic field and in the opposite direction, and then plays the purpose of suppressing noise. However, limited by the real-time performance of the feedback circuit and the accuracy and stability of noise compensation, the compensation effect is not ideal, so most teams combine active and passive noise suppression. Table 6 shows the different environmental noise suppression schemes adopted by different teams.

**Figure 4:**
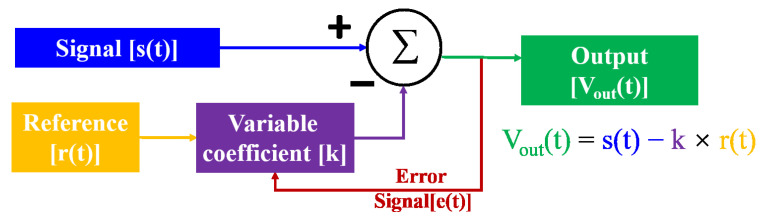
The schematic diagram of adaptive filtering.

Due to the temporal and spatial correlations of magnetic fields, spatial gradient difference [71] is also widely used to reduce the magnetic field noise in addition to the shielding room and Helmholtz coil. A gradiometer in a broad sense is called a synthetic gradiometer and consists of a signal channel and a reference channel. Any magnetic field sensor can be used as signal channel or reference channel. Different research schemes are shown in Table 7. In addition, due to the complex and changeable environmental magnetic field, it is far from enough to only use the hardware gradiometer. An adaptive processing algorithm is also needed [72]. Then the electronic circuit or software algorithm is used for the signal channel and the reference channel to find the time-changing compensation coefficient. An error function is needed for feedback of the results and to guide the optimization of the filtering effect. The schematic diagram of adaptive filtering is shown in Figure 4. No matter what kind of program it is for noise suppression of the environment, it is necessary to combine the characteristics of the environment and the signal to make a comprehensive selection in order to obtain a better effect.

### 2.3. Future Development Trend

Limited by many aspects, the development of neuromuscular magnetic field measurement in China is slow. Clinicians do not know much about sensors, while magnetic sensor researchers focus on sensors and systems. If you want to truly apply the means of magnetic field detection to the clinic, you need to communicate with the doctors and establish a complete chain from the models to the signals and magnetic sensors in order to truly develop the technology. In this paper, the relevant technology, development of sensors and neuromuscular models are combined to provide a comprehensive description, aiming at allowing doctors and researchers to understand each other to promote magnetic field detection to the clinic. It is not only a new research idea that is accordance with the thought that scientific research truly serves the public but also provides references for relevant researchers, and guides researchers to combine medicine with industry and promote progress at the medical level.

For the detection of the neuromuscular magnetic field, a high-sensitivity sensor and a low-noise environment are not enough. Human peripheral nerves [76] consist mainly of 12 pairs of cranial nerves and 31 pairs of spinal nerves. The body has about 639 muscles, which are made up of about 6 billion muscle fibers. Therefore, it is necessary to further improve the spatial resolution at the system level. For multi-channel systems, currently, separate structures are used mainly [77,78]. The channels are separated from each other. Since SQUID is an inductively coupled magnetic flux, crosstalk between channels will occur if the distance between the channels is too close [79]. The suppression of crosstalk will be the focus of the subsequent system. In addition, for neuromuscular detection, portable miniaturized array sensors will become a trend.

In addition, various kinds of sensors were compared in this paper, and the development of SQUID was emphasized. The advantages, disadvantages and necessity of the SQUID sensor in the bio-magnetic field were presented. SQUID will not necessarily be a popular sensor type in the future due to its high cost and large system. However, due to its extremely high sensitivity, it is an inevitable technology to be used in the process of data accumulation and parameter standardization. Atomic sensors are likely to soon become the focus of research in the next few years, and assist SQUID in building some parameters of partial nerve and muscle signals. With the accumulation of data and the standardization of parameters, in the future, equipment miniaturized for portability with the function of real-time monitoring, such as flexible array sensors based on magneto-resistivity, will become a popular hotspot.However, for such sensors, the improvement of their own sensitivity and the integration of the whole system are still the challenges and research emphasis. In addition, no matter what kind of sensors, the readout circuit and the processing of environmental signals are also problems and challenges that need to be solved.

## 3. Application of Neuromuscular Magnetism

Some research groups have made corresponding applications in neuromuscular magnetic detection based on the SQUID multi-channel detection system. Table 8 shows the relevant applications made by different teams in recent years. But they have not really explored the clinical aspect, lacking in-depth research for certain diseases; that is, the cooperation of doctors is needed. For example, acquired inflammatory myopathy has high incidence and a long age span, and its main clinical manifestations are subacute or chronic progressive myasthenia and muscular atrophy, which is one of the most important diseases affecting the quality of life at home and abroad [80]. However, the selection of specific detection sites and patients still needs the cooperation of doctors. In addition, it is difficult to locate the site in clinical myogenic or neurogenic disease [81]. Comprehensive analysis should be carried out based on the characteristics of the cases. And there is an urgent need for more advanced means to solve such problems. MMG has great potential in disease diagnosis, health detection, human–machine interface and rehabilitation [82].

MMG has its unique advantages in disease diagnosis. A team from Shigenori Kawabata in Japan used nerve or muscle magnetic field to examine multiple parts of the human body, demonstrating the advantages of magnetic field detection in disease diagnosis. In 2017, Yoshiaki Adachi introduced the composition of the MSG system and summarized its advantages in multiple bio-magnetic field detection [87]. In 2019, his team used MNG to achieve non-invasive visualization of posterior lumbar nerve root and cauda equina nerve activity [88]. The study data can help establish diagnostic criteria for radiculopathy. In the same year, they used MSG to demonstrate the activity of the brachial plexus and were able to distinguish the conduction pathways following stimulation of the median and ulnar nerves, and further visualized the currents within the axons [89]. In 2020, they used the MNG system to detect the flow of activity after ulnar nerve stimulation and proved that ulnar nerve stimulation was more effective than median nerve stimulation [90]. In 2019, the Adachi team successfully observed the response of the palmar carpal tunnel area and wrist to stimulation [91]; with the progress, artifact removal and source analysis were implemented, and the results showed that MNG was helpful for the diagnosis of various peripheral neuropathy and carpal tunnel syndrome. In 2022, they studied cubital tunnel syndrome by functional imaging of the ulnar nerve of the elbow to rule out false negatives [92].

Compared with MRI, MMG or MSG is functional imaging, so it can test the data for a long time and carry out the functional evolution of the test site, and then infer the possibility of disease at the test site in the future so as to play the purpose of health monitoring. At present, the application of the neuromuscular magnetogram in health monitoring is mainly reflected in the muscle detection of pregnant mothers before and after delivery. In 2004, Curtis L. Lowery’s team used a 151 channel system to detect muscle activity during delivery and successfully predicted the delivery of multiple patients [93]. In 2006, based on previous studies, his team proposed four parameters that could quantify the characteristics of uterine MMG signals and help us better understand the labor process [94]. In 2009, they conducted long-term MMG detection on pregnant mothers, and the detection results proved that MMG has the potential to predict term and preterm birth [95].

Since MMG or MNG can be used for health detection and disease diagnosis, it can be used as a guide for disease rehabilitation. In 1999, Bruno Marcel Mackert used the SQUID system to monitor signals of injured muscles for a long time in vitro, and the results showed that the neuromagnetic detection, quantification and monitoring of in vivo quasi-direct current injury is technically feasible. It also pointed out that the SQUID system can play a role in diagnosing diseases by detecting current changes caused by different depolarization modes in cerebral ischemia cases [96]. Human skeletal muscle is also associated with the occurrence of electrical activities, so MMG is expected to be an auxiliary signal detection means in skeletal muscle physiology research [97]. In 2019, Diana Escalona-Vargas’ team used a multi-channel SQUID array system to characterize the signaling characteristics of the anal levator muscle in pregnant women, demonstrating that MMG provides a novel and innovative tool for studying the female pelvic floor and assessing anal levator function, injury, and rehabilitation [98].

## 4. Neuromuscular Modeling

The signal detection sites carried out by different teams in the worldwide are not the same. Different individuals, and the different nerves or muscles of the same person, are also ever-changing so there are certain problems in the signal interpretation of neuromuscular magnetism. Therefore, building a model based on bone muscle or nerve is a prerequisite for any accurate interpretation of the signal next. Different groups have modeled different parts over time, all based on Maxwell’s equations. Different models used to analyze different muscles or nerves by investigators are shown in Table 9.

The magnetic field generated by a single skeletal muscle fiber was first recorded by Van Egeraat et al. in 1990 [99]. The details of other cellular properties, such as membrane capacitance and intracellular conductivity, were provided using the core conduction model proposed by John P. B et al. in 1985 [100]. It is a model based on a single axon. The model is studied from two perspectives of axial current and radial current inside a single axon, and finally the relationship between the propagating current and the transmembrane potential is obtained.

From Biot–Savart’s law, we know that the magnetic field is inversely proportional to the distance of the source and proportional to the amplitude of the source. From the core conductor model, it can be deduced that the magnitude of the current is equal to the axial derivative of the transmembrane potential divided by the internal resistance per unit length of the axon. Thus, the magnetic field near the nerve is proportional to the derivative of the transmembrane potential. However, when we use sensor detection, the detector is far away from the nerve, so this proportional relationship is no longer true in the actual detection, and the relationship between the magnetic field and the transmembrane potential becomes more complex. In 1985, Woosley and Roth proposed the volume conductor model [101], which took the continuity of the transmembrane potential and the normal component of current density as the boundary condition. They also proposed the expression of potential energy in different media, applied the Fourier transform to define the filter function, and obtained the calculation formula of the current density. Magnetic fields are calculated from the perspective of Biot–Savart’s law and Ampere’s theorem. Although they provide different visual images of the sources and processes that produce magnetic fields, Biot–Savart’s law and Ampere’s theorem produce the same results for all physically measurable quantities. At the same time, the paper also gave some experimental verification, giving different parameters to observe the difference of potential and magnetic field, etc., to study the influence of parameters on the model calculation. Meanwhile, in the same year, Roth and Wikswo made a detection device [102] using a ferrite core, epoxy resin and wire to detect the magnetic field and potential generated by the lobster single axon and verify the accuracy of the model. The results showed that there are differences between theory and practice. Finally, they tried to analyze the sources of the differences in many aspects.

For actual medical detection, the detection of a single axon or single nerve is generally unable to be achieved in vivo, and the clinical detection of nerve bundles composed of multiple nerve fibers can be relatively easy. Therefore, it is more meaningful to study nerve bundles than single axons. In 1991, John. P. Wikswo’s team used the generalized volume conduction model to calculate the composite action potential and current of a nerve bundle [103,104,105]. The effects of the propagation distance and frequency-related conductivity on the composite action signals of various nerve bundles were also studied. At present, the detection of neuromuscular diseases is developing towards the goal of non-invasiveness, but the detection and calculation of the single nerve bundle still fail to meet the demand. The most commonly detected nerves or muscles in clinical tests are superficial, shallower below the surface of the skin. In order to study the mechanism and working mechanism of the nerve control muscle, the detection of a single motor unit compound action potential and potential calculation is used in an in vitro sensor to detect the magnetic field. In 1997, Wikswo’s team [106] developed a simple model to calculate the magnetic field strength of a single moving unit compound action potential at a certain point. Finally, the model was applied to the composite action potentials obtained by SQUID, and information about the distribution of action currents and the anatomical characteristics of individual motor units in muscle bundles could be obtained. In 1998, Tadashi Masuda’s team [107] used the SQUID system to detect magnetic fields in the lateral and medial muscles of three healthy men, and calculated the results using the dipole model and volume conductor model to improve the accuracy of the measurement. With the development and progress of computer technology, the method of using software simulation has become a good means to combine with experimentation. In 2021, Siming Zuo’s team proposed a compact muscle model [82]. COMSOL simulation software was used to establish the model and characterize the action potential of the soleus muscle. Meanwhile, MATLAB was used to derive the relationship between magnetic and electrical signals in physics and mathematics.

Because of the large number and different shapes of human nerves and muscles, it is difficult to propose a universal model, but it is also urgently needed to further promote the development of magnetic field detection in clinical applications.

**Table 9:** Different models used to analyze muscles or nerves.

Ref.	Model	Object	Sensor
[99]	Core Conductor Model	a Single Skeletal Muscle Fiber from Frog Gastrocnemius	Toroidal Pickup Coil
[100]	Core Conductor Model	a Single Nerve Axon of the Crayfish	SQUID
[101]	Volume Conductor Model	an Isolated Nerve Axon	/
[102]	Volume Conductor Model	Medial Giant Axon of a Crayfish	Toroidal Pickup Coil
[103,104,105]	Volume Conductor Model	Peripheral Nerve Bundle	Toroidal Pickup Coil
[106]	Tripole Model, Current Element Model	Single Motor Unit	SQUID
[107]	Dipole Model, Volume Vonductor Model	Vastus Lateralis and Vastus Medialis	SQUID
[82]	Compact Muscle Model	Soleus Muscle	/

## 5. Conclusions

So far, SQUID is the sensor with the highest sensitivity for detecting the neuromuscular magnetic field, and its application advantages in clinical detection are not obvious. However, SQUID is the best choice for basic research on neuromuscular pathogenesis and the current propagation mechanism. In this paper, the sensor-related technologies, including devices, circuits, the relevant environmental testing and noise suppression technology in the field of weak magnetic detection are introduced, and the future development trend of neuromuscular magnetic field detection is described. Finally, the status quo and development of neuromuscular magnetic field detection at home and abroad are summarized and described from the aspects of application and modeling. Due to the characteristics of the magnetic field, MMG will play a significant role in medical diagnosis, thus improving the level of human health.

As the development of the technologies, the miniaturization of sensors has become an increasingly important goal for investigators. For MMG measurement, reluctance sensors will become one of the sensors that can realize miniaturization and array to serve human demand. And it is crucial to improve the sensitivity of magneto-resistive sensors. For MNG measurement, OPM or other atomic magnetometers will become one of the sensors that can realize miniaturization. Nevertheless, no matter what kind of sensors, before the system is applied in clinical diagnosis, in order to gradually apply the means of magnetic detection to real life, it needs abundant experiments and large sample data collection. Meanwhile, the establishment of corresponding detection standards is an important next task. The acquisition and analysis of large sample data using SQUID is also the only way for development due to its sensitivity and bandwidth and some other advantages.

## Figures and Tables

**Table 1:** Comparison of several detection methods.

	MMG	ECG	MRI
Magnetic Field Signal	yes	electronic signal	strong magnetic field as excitation
Invasive/noninvasive	noninvasive	invasive	noninvasive
Muscle activity	yes	yes	tissue imaging
Frequency	DC-MHz	low frequency	/
Space Resolution	mm-cm	mm	mm
Time Resolution	ms	ms	s-min
Magnetic Field Signal	yes	electronic signal	strong magnetic field as excitation
Activity positioning and accuracy	precision	inaccuracy	tissue imaging
Activity latent detection	precision	precision, but depend on experience	tissue imaging
Peripheral nerve function detection	yes	yes	tissue imaging
Nerve conduction	yes	yes	tissue imaging

**Table 2:** Preparation of Josephson junctions by different research teams.

Ref.	Institution	Size	Jc (kA/cm${}^{2}$)
[35]	MIT LL	d = 200 nm	50
[36]	AIST	1 × 1 μm${}^{2}$	10
[37]	NIST	d = 2.7 μm	4.9
[38]	SIMIT	d = 0.5 μm	15

**Table 3:** Research status of different SQUID sensors.

Insitution	Sensor	Sensitivity	Noise
NIM [39]	Current sensor	2.4 μA/ϕ${}_{0}$	1 pA/$\sqrt{\mathrm{Hz}}$
Star Cryoelectronics [40]	Current sensor	0.2 μA/ϕ${}_{0}$	0.6 pA/$\sqrt{\mathrm{Hz}}$
PTB [41]	Current sensor	22.5 μA/ϕ${}_{0}$	9 pA/$\sqrt{\mathrm{Hz}}$
NIST [42]	Current sensor	8.4 μA/ϕ${}_{0}$	1.6 pA/$\sqrt{\mathrm{Hz}}$
Heidelberg University [43]	Current sensor	12.7 μA/ϕ${}_{0}$	2.9 pA/$\sqrt{\mathrm{Hz}}$
SIMIT [44]	Current sensor	25 μA/ϕ${}_{0}$	7 pA/$\sqrt{\mathrm{Hz}}$
KRISS [45]	Magnetometer	1 mV/ϕ${}_{0}$	1.5 μϕ${}_{0}$/$\sqrt{\mathrm{Hz}}$
IPHT [46]	Magnetometer	0.4 μA/ϕ${}_{0}$	0.1 fT/$\sqrt{\mathrm{Hz}}$ @ white noise
NBU [20]	Gradiometer	0.54 nT/ϕ${}_{0}$	3.5 fT/$\sqrt{\mathrm{Hz}}$

**Table 4:** Performance contributions of SQUID readout circuits based on system-level applications.

Institution	Bandwidth (MHz)	Dynamic Range (dB)	Slew Rate (mT/s)
SIMIT [54]	0.12	160	3
Jülich [55]	0.02	130	2
Tristan Technologies [56]	0.05	160	1.1
SUSTERA [57]	0.1	99	10
CSIRO [58]	1	110	2.66
Supracon AG [59]	0.1	165	5–10

**Table 5:** Passive shielding schemes developed by different research teamsgroups.

Model	Construction	Remanence	Shielding Factor @ 1 Hz (SE = 20 × log (Bo/Bin))
BMSR-2 [62]	7 layers permalloy + 1 layer aluminum	0.5 nT	10${}^{8}$
VAC [63]	7 layers soft magnet nickel alloy + 1 layer aluminum	0.01 nT	10${}^{6}$
IMECO [64]	5 layers soft magnet nickel alloy + 1 layer aluminum	<0.5 nT	10${}^{5}$
COSMOS [65]	4 layers permalloy + 1 layer aluminum	/	4.2 × 10${}^{5}$

**Table 6:** Active and passive environmental noise compensation schemes of different groups.

Ref.	Institution	Construction	Results
[66]	Beihang University	Shielding room + Axial coils	>32dB
[67]	Aalto University	Shielding room + Triaxial coils	22 dB
[68]	The University of Nottingham	Shielding room + Biplanar coils	40 dB
[69]	Warsaw University of Technology	Shielding room + Triaxial coils	32–38 dB
[70]	The University of Nottingham	Shielding room + Matrix coils	Field Changes < ±1 nT

**Table 7:** Comparison of different gradiometer suppression schemes.

Institution	Construction	Performance	Environment
Epilepsy and Brain Mapping Center [71]	Magnetometer + Gradiometer	<10 fT/$\sqrt{\mathrm{Hz}}$	MSR
SIMIT [72]	Magnetometer + Gradiometer	noise rejection 100 dB	No Shielding
Sharif university of Technology [73]	Magnetometer + Magnetometer	<10${}^{-5}$ϕ${}_{0}$/$\sqrt{\mathrm{Hz}}$	MSR
SIMIT [74]	Full Tensor + Gradiometer	SNR 27.7 dB	No Shielding
Boston Children’s Hospital [75]	Magnetometer + Magnetometer	<10 fT/$\sqrt{\mathrm{Hz}}$	MSR

**Table 8:** Application progress of neuromuscular magnetism in different groups.

Ref-Channel	Experiment Environment	System Noise	Signal
Ref. [83]-275	No shielding room + three-order gradiometer	4-7 fT/$\sqrt{\mathrm{Hz}}$	Skeletal muscles of the hand and muscles of the lower back
Ref. [84]-304	Shielding room	2.3 fT/$\sqrt{\mathrm{Hz}}$ @ 1 kHz	Median nerve
Ref. [85]-151	Shielding room	5 fT/$\sqrt{\mathrm{Hz}}$	Levator muscle
Ref. [86]-142	Shielding room	3–4 fT/$\sqrt{\mathrm{Hz}}$	Nervi spinalis
